# Biosorption optimization, characterization, immobilization and application of *Gelidium amansii* biomass for complete Pb^2+^ removal from aqueous solutions

**DOI:** 10.1038/s41598-018-31660-7

**Published:** 2018-09-07

**Authors:** Noura El-Ahmady El-Naggar, Ragaa A. Hamouda, Ibrahim E. Mousa, Marwa S. Abdel-Hamid, Nashwa H. Rabei

**Affiliations:** 1 Department of Bioprocess Development, Genetic Engineering and Biotechnology Research Institute, City of Scientific Research and Technological Applications, Alexandria, Egypt; 2 Microbial Biotechnology Department, Genetic Engineering and Biotechnology Research Institute, University of Sadat City, 22857 Menoufyia Governorate, Egypt; 3 Environmental Biotechnology Department, Genetic Engineering and Biotechnology Research Institute (GEBRI), University of Sadat City, 22857 Menoufyia Governorate, Egypt

## Abstract

Lead (Pb^2+^) is among the most toxic heavy metals even in low concentration and cause toxicity to human’s health and other forms of life. It is released into the environment through different industrial activities. The biosorption of Pb^2+^ from aqueous solutions by biomass of commonly available, marine alga *Gelidium amansii* was studied. The effects of different variables on Pb^2+^ removal were estimated by a two-level Plackett–Burman factorial design to determine the most significant variables affecting Pb^2+^ removal % from aqueous solutions. Initial pH, Pb^2+^ concentration and temperature were the most significant factors affecting Pb^2+^ removal chosen for further optimization using rotatable central composite design. The maximum removal percentage (100%) of Pb^2+^ from aqueous solution by *Gelidium amansii* biomass was found under the optimum conditions: initial Pb^2+^ concentration of 200 mg/L, temperature 45 °C, pH 4.5, *Gelidium amansii* biomass of 1 g/L and contact time of 60 minutes at static condition. FTIR analysis of algal biomass revealed the presence of carbonyl, methylene, phosphate, carbonate and phenolic groups, which are involved in the Pb^2+^ ions biosorption process. SEM analysis demonstrates the ability of *Gelidium amansii* biomass to adsorb and removes Pb^2+^ from aqueous solution. EDS analysis shows the additional optical absorption peak corresponding to the Pb^2+^ which confirms the involvement of *Gelidium amansii* biomass in the adsorption of Pb^2+^ ions from aqueous solution. Immobilized *Gelidium amansii* biomass was effective in Pb^2+^ removal **(**100%**)** from aqueous solution at an initial concentration of 200 mg/L for 3 h. In conclusion, it is demonstrated that the red marine alga *Gelidium amansii* biomass is a promising, efficient, ecofriendly, cost-effective and biodegradable biosorbent for the removal of Pb^2+^ from the environment and wastewater effluents.

## Introduction

Heavy metals are the main group of inorganic contaminants. Main sources of heavy metals contamination include agricultural chemicals (pesticides, fertilizers) and industrial activities including plating, petroleum refining, mining activities, smelting industries, car exhausts, battery manufacturing and pigments. Industrial activities and agricultural chemicals often discharge wastes containing heavy metals that flow into streams, lakes, ground water and rivers. The presence of heavy metals in aqueous water streams is hazardous to the environment, poses a potential human health risks and causes harmful effects to living organisms in water and also to the consumers of them^1,2^.

Lead is among the most toxic heavy metals affecting the environment^3^. Lead pollution results from textile dyeing, pigments, ceramic and glass industries, petroleum refining, metal plating and finishing, battery manufacturing and mining operations^1,4^. Lead even at low concentrations can be hazardous and cause toxicity to humans and other forms of life. The USA environmental protection agency regulations for drinking water limits lead in drinking water to 0.015 mg/L. While a drinking-water guideline value for lead of 0.01 mg/L has been established by WHO^5^. According to India standard drinking water specification, highest desirable limit of lead in drinking water is 0.05 ppm (0.05 mg/L)^6^. The toxicity of metal ions is owing to their ability to bind with protein molecules and prevent replication of DNA and thus subsequent cell division^7^. Lead accumulates mainly in bones, brain, kidney and muscles and the increase in lead concentration may cause many serious disorders like anaemia, kidney and liver diseases, gastrointestinal damage, nervous disorders and sickness even death^8,9^. It is therefore, essential to remove Pb(II) from wastewater before disposal.

Conventional methods applied for lead removal from industrial waste waters and aqueous solutions include coagulation and precipitation, electrochemical treatment, ion exchange, chemical oxidation or reduction, evaporation, electroplating adsorption, and membrane separation. However, these methods have several disadvantages, such as generation of toxic waste products, too expensive, not always effective for metals with low concentrations, high reagent and energy requirements^10–12^. Adsorption by activated carbon is the most efficient classical method, but the cost of its production is expensive and it cannot be recycled^13^.

Consequently, it is urgent to find cost-effective alternative technologies to remove heavy metal ions from waste water^14,15^. Biosorption is effective biological treatment of wastewater that utilizes low cost biosorbents for the removal of toxic heavy metals^16^. Biosorption could be considered as a promising alternative technique for heavy metal ions removal^10^ as it offers many advantages over traditional treatment methods including cost-effectiveness, high metal binding ability, high efficiency in diluted effluents, environmentally friendly^17^ and regeneration of biosorbent with possibility of metal recovery.

Biosorbents for lead removal include fungi^18^, bacteria^19^ and algae biomass^20,21^. Algae proved to possess high metal binding capacities^22^ because of the presence of proteins, polysaccharides or lipid on their cell walls surfaces containing some functional groups such as carboxyl, hydroxyl, amino and sulphate, which can act as binding sites for metals^23,24^.

Heavy metals biosorption is affected by many environmental variables such as temperature, pH, ionic strength, etc. A statistical approach has been employed in the present study for which a Plackett–Burman design was used for identifying significant variables influencing the biosorption of Pb^2+^ from aqueous solutions by *Gelidium amansii*. The levels of the significant variables and the interaction effects between various variables which influence the biosorption of Pb^2+^ were further analyzed and optimized using rotatable central composite design (RCCD).

The aim of the study was to investigate the efficiency of *Gelidium amansii* as a cost effective biosorbent for Pb^2+^ removal from aqueous solutions, the statistical optimization for Pb^2+^ removal, biomass characterization before and after Pb^2+^ biosorption using SEM, FTIR and EDS, in addition to *Gelidium amansii* immobilization in sodium alginate beads and its application in Pb^2+^ removal.

## Results and Discussion

Heavy metals biosorption from aqueous solutions can be considered a promising technique in the treatment of wastewater. It is based on the ability of biological materials (which can include living or dead microorganisms and their components, seaweeds, etc.) to collect heavy metals ions from wastewater through physicochemical absorption or metabolically mediated pathways of uptake^25,26^. Biosorption is determined by equilibrium, it is largely influenced by the concentration of biomass, pH and the interaction between various metals ions^8^. A number of physico-chemical factors determine overall biosorption performance^27^. Since the main mechanism of biosorption was found to be ion exchange, protons compete with metal cations for the binding sites and for this reason; pH is the most important process parameter which influences the availability of the site to the sorbate^28^. The other factors important in biosorption include the biosorbent nature and the availability of binding sites (type and the concentration of the biomass)^29^; initial heavy metal concentration which when increased increases the quantity of biosorbed heavy metals per unit weight of the biomass, but decreases removal efficiency; temperature usually enhances heavy metals removal when increased by increasing surface activity and kinetic energy of the adsorbate^27^. The effects of biomass concentrations, heavy metal concentrations, temperature, pH, agitation/static and contact time on the biosorption of Pb^2+^ have been studied.

### Screening of significant variables affecting the Pb^2+^ removal % by *Gelidium amansii* biomass using Plackett–Burman design

The effect of the six variables considered in this study (namely: contact time, initial Pb^2+^ ions concentration, pH, temperature, biomass and agitation/static) on Pb^2+^ removal % was statistically analyzed using Plackett–Burman Design (PBD). The *Gelidium amansii* biomass was dried in oven at 70 °C for 72 hrs, and then milled with a blender, sieved to get particle with the size pass through a laboratory test sieve Endecotts/ Ltd., London, England, with mesh size of 125 µm (Supplementary Fig. S1).

The design matrix of the Plackett-Burman used to determine the most significant variables affecting Pb^2+^ removal % from aqueous solutions using *Gelidium amansii* biomass is shown in Table 1. The experiment was conducted in 12 runs. Table 1 shows the levels of coded and actual values of the tested independent variables and the Pb^2+^ removal % in each run. The data listed in Table 1 indicated a variation on lead removal %, from 92.52 to 99.69, in the 12 trials. This variation suggested that the process optimization was important for improving the removal efficiency of lead to attain maximum Pb^2+^ removal. Results showed the highest lead removal % (99.69%) in run no. 7. The relationship between Pb^2+^ removal % and the independent variables was analyzed with regard to their effects on Pb^2+^ removal % using a Plackett–Burman design (Table 2). The coefficient of each factor represents the effect extent of this factor on Pb^2+^ removal. Analysis of the regression coefficients of the six factors (Table 2) showed that Pb^2+^ concentration (B) and temperature (D) with coefficient value 0.67 and 0.86 and percent of contribution 15.80 and 20.28%; respectively) had positive effects on lead removal % which means that the increase in temperature and Pb^2+^ concentration could exert positive effect on Pb^2+^ removal. Where, contact time (A), pH (C), biomass concentration (E) and agitation-static (F) (with coefficient value −0.07, −2.06, −0.28 and −0.30 and percent of contribution 1.65%, 48.58%, 6.60% and 7.08%; respectively) had negative effects which means that the decrease in contact time, pH, biomass concentration and agitation/ static levels could exert positive effect on Pb^2+^ removal.

**Table 1:** Twelve-trials Plackett–Burman experimental design for evaluation of independent variables with coded and actual levels along with the observed and predicted values of lead (Pb^2+^) removal by *Gelidium amansii* biomass.

Run no.	Coded and actual levels of independent variables												Pb^2+^ removal (%)		Residuals
	Contact time (minutes)		Pb^2+^ concentration (mg/L)		pH		Temperature (°C)		Biomass (g/L)		Agitation -Static		Actual value	Predicted value	
1	−1	60	−1	25	−1	4	1	50	1	4	1	Agitation	97.34	97.14	0.20
2	−1	60	−1	25	1	7	1	50	1	4	−1	Static	93.98	93.62	0.36
3	−1	60	1	200	1	7	1	50	−1	1	1	Agitation	94.32	94.90	−0.58
4	1	180	−1	25	1	7	1	50	−1	1	1	Agitation	93.45	93.43	0.02
5	1	180	−1	25	−1	4	−1	25	1	4	1	Agitation	94.95	95.27	−0.32
6	−1	60	1	200	−1	4	−1	25	−1	1	1	Agitation	97.93	97.30	0.63
7	1	180	1	200	−1	4	1	50	−1	1	−1	Static	99.69	99.49	0.20
8	1	180	1	200	1	7	−1	25	1	4	1	Agitation	92.52	92.47	0.05
9	−1	60	−1	25	−1	4	−1	25	−1	1	−1	Static	96.06	96.57	−0.51
10	1	180	−1	25	1	7	−1	25	−1	1	−1	Static	92.55	92.30	0.25
11	1	180	1	200	−1	4	1	50	1	4	−1	Static	98.73	98.93	−0.20
12	−1	60	1	200	1	7	−1	25	1	4	−1	Static	93.13	93.22	−0.09

The −1 sign correspond to the minimum value and the +1 sign correspond to the maximum value of the input parameter range

**Table 2:** Regression statistics and analysis of variance (ANOVA) for the experimental results of Plackett-Burman design used for Pb^2+^ removal by *Gelidium amansii* biomass.

Source	*df*	Coefficient	Effect	Contribution %	*t -*Stat	*P*-value	Confidence Level (%)
Intercept		95.39			619.14	2.08E-13	100
Contact time (A)	1	−0.07	−0.14	1.65	−0.47	0.658	34.2
Pd concentration (B)	1	0.67	1.34	15.80	4.32	0.008*	99.2
pH (C)	1	−2.06	−4.12	48.58	−13.39	0.000042*	99.9958
Temperature (D)	1	0.86	1.72	20.28	5.61	0.0025*	99.75
Biomass (E)	1	−0.28	−0.56	6.60	−1.81	0.130	87
Agitation –Static (F)	1	−0.30	−0.6	7.08	−1.96	0.107	89.3
R	0.9896	R-Sq(adj)	0.954	PRESS	8.203		
R^2^	0.979	R-Sq(pred)	0.8809				
	***df***	***SS***	***MS***	***F***	***Significance F***		
Regression	6	67.425	11.237	39.45	0.00047		
Residual error	5	1.424	0.285				
Total	11	68.849					

*Significant values, *df*: Degree of freedom, *F*: Fishers’s function, *P*: Level of significance.

**Figure 1:**
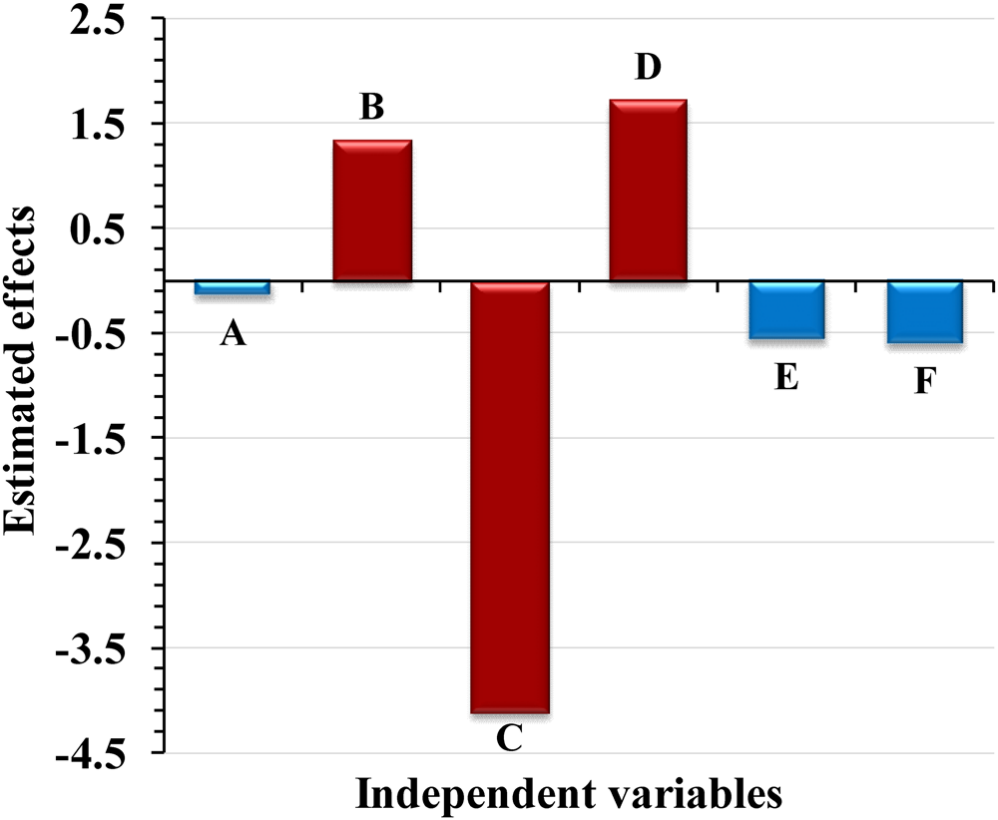
Estimated effects of independent variables on Pb^2+^ removal by *Gelidium amansii* biomass using Plackett-Burman design “the red color represents the most significant independent variables affecting Pb^2+^ removal”.

Table 2 and Fig. 1 show the estimated effect of each variable on Pb^2+^ removal %. The large effect, either negative or positive, indicates that the variable has a large impact on Pb^2+^ removal, whereas the near zero effect means that the variable has little or no effect. The results indicated that the high levels of initial Pb^2+^ ion concentration and temperature positively affected on lead removal %. Whereas, high levels of the other four variables (pH, contact time, biomass and agitation) negatively affected on lead removal %. The contact time (A), biomass (E) and agitation/static (F) are three insignificant variables with lower effects (−0.14, −0.56 and −0.6; respectively). Pareto chart (Supplementary Fig. S2) showed that pH (C) was the most significant variable affecting Pb^2+^ removal (48.58%) by *Gelidium amansii*, followed by temperature (D) (20.28%), Pb^2+^ concentration (B) (15.80%), then agitation/static (F), biomass (E) and contact time (A); respectively.

### The normal probability plot (NPP) of the residuals

**Figure 2:**
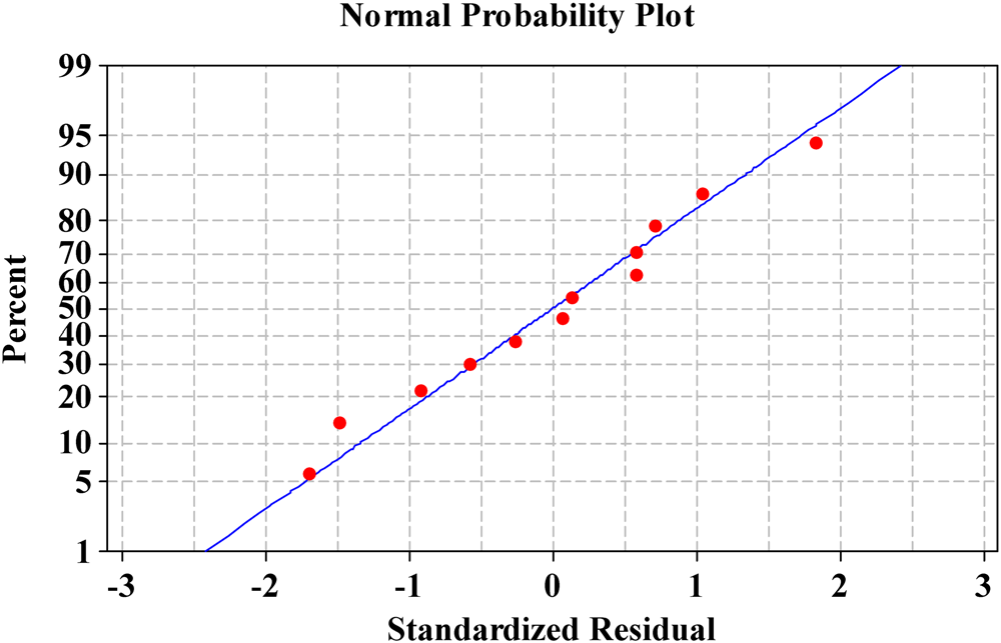
The normal probability plot of the residuals for Pb^2+^ removal by *Gelidium amansii* biomass determined by the first-order polynomial equation.

A normal probability plot is a plot represents the normal distribution of the residuals to check the adequacy of the model^30^. The residuals are the variation between the experimental values of the responses and the values that expected by the theoretical model. A small residual values shows that model prediction is very accurate^31^. Figure 2 shows NPP of the residuals plotted against the expected values of the model. The data points (the residuals from the fitted model) are found close to the diagonal line for Pb^2+^ removal %, however the data appear to be normally distributed and signifying the validity of the model.

The ANOVA of the Plackett-Burman design demonstrated that the model was highly significant as was evident from the Fisher’s *F*-test (39.45) with a very low *P*-value (0.00047) and the *t*-Stat (619.14). The variable with confidence level above 95% is considered as significant parameter. The factors evidencing *P*-values of less than 0.05 were considered to have significant effects on the Pb^2+^ removal. The *t*-values and *P*-values were estimated for each independent variable as shown in Table 2 and were used as a tool to check the significance of each parameter. The results in Table 2 showed that the most significant variables which affecting in lead removal by *Gelidium amansii* with confidence level above 95% was pH (C) having a probability value of 0.000042 then temperature (D) and Pb^2+^ concentration (B) with probability values of 0.0025 and 0.008; respectively. While, contact time, biomass and agitation, with confidence levels below 95%, were considered insignificant.

The fit of the model was checked by the determination of coefficient (R^2^). The R^2^ value is always between 0 and 1. If the value of R^2^ is close to 1, the model is stronger and better to predict the response. In the present study, the R^2^ value is 0.979 indicated that up to 97.9% variability in lead removal % could be calculated by the model and only 2.1% of the total variability in lead removal % is not explained by the model. In addition, the value of adjusted determination coefficient (adjusted R^2^ = 0.954) is also very high, showing a high significance of the model (Table 2). Therefore, R^2^ and R^2^ adj emphasized that the model is highly significant and suitable to explain of the relationship between the selected variables and the Pb^2+^ removal %. The value of predicted R^2^ is also high to support a high significance and accuracy of the model. The predicted R^2^ value obtained is 0.8809, indicating that the model does not explain only 11.91% of the total variations. This also revealed that predicted R^2^ of 0.8809, for the Pb^2+^ removal by *Gelidium amansii*, is in reasonable agreement with the adjusted R^2^ of 0.954 indicating that a good agreement between the experimental and predicted values of Pb^2+^ removal %.

By applying multiple regression analysis on the experimental data, the experimental results of Plackett-Burman design were fitted with the first-order polynomial equation which represent the Pb^2+^ removal % as a function of the contact time, Pb^2+^ concentration, pH, temperature, biomass and agitation–static.

1 $$\begin{array}{rcl}{{\rm{Pb}}}^{2+}{\rm{removal}}\,( \% ) & = & {\rm{95.39}}-{\rm{0.07}}\,{\rm{contact}}\,{\rm{time}}\,(\mathrm{minutes})\\  &  & +0{{\rm{.67Pb}}}^{2+}{\rm{concentration}}({\rm{mg}}/{\rm{L}})-{\rm{2.06}}\,\mathrm{pH}+0.86\,{\rm{temperature}}\,(^\circ {\rm{C}})\\  &  & -{\rm{0}}{\rm{.28biomass}}\,({\rm{g}}/{\rm{L}})-{\rm{0.30}}\,\mathrm{agitation}\mbox{--}\mathrm{static}\end{array}$$

In a confirmatory experiment, to evaluate the accuracy of Plackett-Burman, the conditions which expected to be optimum for maximum Pb^2+^ removal by *Gelidium amansii* from aqueous solutions were contact time of 60 minutes, initial Pb^2+^ concentration of 200 mg/L, pH 4, temperature 50 °C, *Gelidium amansii* biomass of 1 g/L at static condition. Under these conditions, the maximum removal percentage of Pb^2+^ was 94.3% which is higher than the removal percentage of Pb^2+^ obtained before applying Plackett Burman (45.9%) by 2.05 times.

In the present study, the agitation is non-significant factors (*P*-value = 0.107). Our results are in agreement with those reported by Saraf and Vaidya^32^ in that the agitation had a negative effect on the biosorption. This may be due to the swelling behaviour of the biomass particles of the red algae due to the presence of a high proportion of carrageenan constituting up to 75% of the dry weight of the biomass, making it suspended in the solutions. Whereas, Tahir *et al*.^33^ reported that agitation enhances biosorption and facilitates proper contact between the metal ions in solution and the biomass-binding sites and thereby promotes effective transfer of sorbate ions to the sorbent sites.

### Statistical optimization of Pb^2+^ removal by *Gelidium amansii* using rotatable central composite design (RCCD)

On the basis of *P-*values (Table 2), initial pH (X_1_), Pb^2+^ concentration (X_2_) and temperature (X_3_) were chosen for further optimization using rotatable central composite design, where these variables were the most significant factors affecting Pb^2+^ removal. The RCCD had six axial points, eight factorial and six center points resulting in a total of 20 experiments used to optimize the chosen variables, all in three replicates. The six replicates at the centre points were conducted to determine the experimental errors. A design matrix that contains the three variables, their coded and actual levels and the responses which are percentage removal of Pb^2+^ was displayed in Table 3. Contact time, biomass and agitation–static which exerted a negative effect on Pb^2+^ removal and are insignificant variables were maintained in all trials at their low levels of Placket-Burman design for further optimization by RCCD.

**Table 3:** Rotatable central composite design representing Pb^2+^ removal % by *Gelidium amansii* as influenced by pH (X_1_), Pb^2+^ concentration (X_2_) and temperature (X_3_) along with the predicted Pb^2+^ removal % and residuals and the actual factors levels corresponding to the coded factors levels.

Std	Run	Type	Variables			Pb^2+^ removal (%)				Residuals
			X_1_	X_2_	X_3_	Experimental		Predicted		
4	1	Factorial	1	1	−1	92.64		91.95		0.69
1	2	Factorial	−1	−1	−1	91.75		91.76		−0.01
19	3	Center	0	0	0	100		99.80		0.20
6	4	Factorial	1	−1	1	85.88		86.23		−0.35
14	5	Axial	0	0	1.68	87.23		86.38		0.85
13	6	Axial	0	0	−1.68	88.1		88.57		−0.47
9	7	Axial	−1.68	0	0	92.07		91.62		0.45
11	8	Axial	0	−1.68	0	94.4		93.63		0.77
2	9	Factorial	1	−1	−1	94.73		94.92		−0.19
8	10	Factorial	1	1	1	92.17		92.43		−0.26
20	11	Center	0	0	0	99.99		99.80		0.19
7	12	Factorial	−1	1	1	93.78		93.86		−0.08
5	13	Factorial	−1	−1	1	87.73		88.69		−0.96
15	14	Center	0	0	0	99.99		99.80		0.19
17	15	Center	0	0	0	100		99.80		0.20
16	16	Center	0	0	0	98.75		99.80		−1.05
12	17	Axial	0	1.68	0	95.08		95.47		−0.39
18	18	Center	0	0	0	99.99		99.80		0.19
10	19	Axial	1.68	0	0	93.01		93.07		−0.06
3	20	Factorial	−1	1	−1	87.85		87.77		0.08
**Variable**				**Variable code**		**Coded and actual levels**				
						**−1.68**	**−1**	**0**	**1**	**+1.68**
pH				X_1_		1.98	3	4.5	6	7.02
Lead (Pb^2+^) concentration (mg/L)				X_2_		31.82	100	200	300	368.18
Temperature (°C)				X_3_		19.77	30	45	60	70.23

Experimental and predicted Pb^2+^ percentage removals for the twenty trials of the employed RCCD matrix are presented in Table 3. Depending on the differences in the three independent variables, the results show variation in the percentage of Pb^2+^ removal. Pb^2+^ removal ranged from 85.88 to 100%. The highest levels of Pb^2+^ removal were obtained in run no. 3 and 15 with value of 100%, where pH is 4.5, Pb^2+^ concentration is 200 mg/L and temperature is 45 °C. While the minimum Pb^2+^ removal was observed in run number 4 with value of pH is 6, Pb^2+^ concentration is 100 mg/L, and temperature is 60 °C.

### Multiple regression analysis and ANOVA

The multiple regression analysis of the model and the analysis of variance (ANOVA) are presented in Tables 4 and 5. A regression model with a determination coefficient (R^2^) value higher than 0.9 having a very high correlation^34^. R^2^ value should not be less than 0.75 until the model is appropriate^35^. However, Koocheki *et al*.^36^ assumed that a large R^2^ value does not always mean that the regression model is good and such conclusion can only be made based on a high value of adjusted R^2^. The present R^2^ and adjusted R^2^ values for removal of Pb^2+^ using *Gelidium amansii* biosorbent were found to be 0.9891 and 0.9792; respectively indicating the fitness of the model for the experimental data. The determination coefficient (R^2^ = 0.9891) indicated that the model cannot explain only 1.09% of the total variations and 98.91% of Pb^2+^ removal variations can be described by the selected model. In addition, the adjusted determination coefficient (R^2^ adj = 0.9792) is also very high, indicating a high significance of the model, which indicated a good agreement between the experimental and predicted values of Pb^2+^ removal. Predicted R^2^ is a measure of how model significance in predicting the response value. The predicted R^2^ (R^2^ pred = 0.9340) is also high enough to indicate the high significance of the model. The adjusted and predicted R-squared values should be within 20% of each other to be in good agreement^37^. In our study, the predicted R^2^ is 0.9340, revealed that it is in a reasonable agreement with the adjusted R^2^ value of 0.9792. This indicated a good agreement between the observed and predicted values and showing that the model offers 93.40% variability in Pb^2+^ removal prediction in the range of experimental variables. Thus the model is adequate for prediction in the range of the experimental variables. The negative coefficient values indicate that linear, mutual interactions or quadratic effects of the variables negatively affect Pb^2+^ removal % by *Gelidium amansii* (inverse relationship between the factor (s) and the biosorption percentage), whereas positive coefficient values mean that the variables increase Pb^2+^ removal % by *Gelidium amansii* in the tested range of the experimental variables (Table 4). Interactions between two factors could appear as an antagonistic effect (negative coefficient) or a synergistic effect (positive coefficient). A low value of the coefficient of variation % (CV = 0.74%) shows a better precision and reliability of the experiments^38^. Adequate precision value of the present model is 27.62 and this value suggests that the model can be used to navigate the design space. PRESS value in the current study is 29.16. Standard deviation and mean value are 0.69 and 93.76; respectively (Table 4).

**Table 4:** Regression statistics of rotatable central composite design, regression coefficients of second order polynomial model for Pb^2+^ removal % by *Gelidium amansii* biomass.

Factor	Coefficient estimate	Standard error	95% CI Low	95% CI High
Intercept	99.80	0.28	99.17	100.43
X_1_ – (pH)	0.43	0.19	0.01	0.85
X_2_ – (Pb^2+^ concentration, mg/L)	0.55	0.19	0.13	0.97
X_3_ – (Temperature,°C)	−0.65	0.19	−1.07	−0.23
X_1_X_2_	0.26	0.25	−0.29	0.80
X_1_X_3_	−1.40	0.25	−1.95	−0.86
X_2_X_3_	2.29	0.25	1.74	2.84
X_1_^2^	−2.63	0.18	−3.04	−2.23
X_2_^2^	−1.86	0.18	−2.26	−1.45
X_3_^2^	−4.36	0.18	−4.76	−3.95
Std. Dev.	0.69	R-Squared		0.9891
Mean	93.76	Adj R-Squared		0.9792
C.V.%	0.74	Pred R-Squared		0.9340
PRESS	29.16	Adeq Precision		27.62

C.V: Coefficient of variation, PRESS: sum of squares of prediction error.

**Table 5:** Analysis of variance (ANOVA) for rotatable central composite design results used for Pb^2+^ removal % by *Gelidium amansii* biomass.

Source	Sum of Squares	*df*	Mean Square	*F-*value	*P-*value *P*rob > *F*
Model	436.79	9	48.53	100.63	<0.0001*
X_1_ – (pH)	2.54	1	2.54	5.27	0.0446
X_2_ – (Pb^2+^ concentration, mg/L)	4.11	1	4.11	8.53	0.0153*
X_3_ – (Temperature,°C)	5.77	1	5.77	11.95	0.0061*
X_1_X_2_	0.53	1	0.53	1.09	0.3212
X_1_X_3_	15.76	1	15.76	32.69	0.0002*
X_2_X_3_	42.00	1	42.00	87.08	<0.0001*
X_1_^2^	99.95	1	99.95	207.23	<0.0001*
X_2_^2^	49.63	1	49.63	102.90	<0.0001*
X_3_^2^	273.58	1	273.58	567.25	<0.0001*
Residual	4.82	10	0.48		
Lack of Fit	3.53	5	0.71	2.74	0.1464
Pure Error	1.29	5	0.26		
Cor Total	441.61	19			

*Significant values, *df*: Degree of freedom, *F*: Fishers’s function, *P*: Level of significance.

The ANOVA results (Table 5) demonstrates that the model is highly significant as evident from the Fisher’s *F* test (*F* value of 100.63) with a very low probability value (*P-*value less than 0.0001). Values of Prob > *F* less than 0.05 indicate that model terms are significant. The Lack of Fit *F*-value of 2.74 is not significant as the *P*‒value is >0.05 (0.1464). Therefore, the high value of adjusted R^2^ of the model, non-significance lack-of-fit, high *F*‒value, low standard deviation and coefficient of variance, low PRESS value and high adequate precision indicate high precision and validity of the model used in predicting the Pb^2+^ removal efficiency using *Gelidium amansii* biomass.

The significance of each coefficient was determined by the probability values (*P*-value) and *F*-value which are listed in Table 5. The coefficient is significant if the *F*-value is large and *P* < 0.05. Based on *P*‒values and *F*‒values, it can be seen from the degree of significance that the linear coefficients of initial pH (X_1_), Pb^2+^ concentration (X_2_) and temperature (X_3_), interaction between X_1_X_3_ and X_2_X_3_, quadratic effect of X_1_, X_2_ and X_3_ are significant as can be seen by the *F*‒values of 5.27, 8.53, 11.95, 32.69, 87.08, 207.23, 102.90, 567.25; respectively, as well as *P*‒values of 0.0446, 0.0153, 0.0061, 0.0002, <0.0001, <0.0001, <0.0001, <0.0001; respectively. Furthermore, *P*‒values of the coefficients suggest that among the three variables studied, the interaction between X_2_ and X_3_ had a very significant effect on Pb^2+^ removal by *Gelidium amansii* with *F*‒value of 87.08 and a probability value of <0.0001, indicating that <99.99% of the model affected by Pb^2+^ concentration (X_2_) and temperature (X_3_). On the other hand, the interaction effect between initial pH (X_1_), Pb^2+^ concentration (X_2_) is not significant model term that not contribute to the response (Pb^2+^ removal).

The fit summary results (Supplementary Table S1) showed that, the quadratic model is a highly significant model fitting the RCCD used for Pb^2+^ removal by *Gelidium amansii* with a very low probability value (*P*‒value < 0.0001), also lack of fit *F*‒value 2.74 (the lack of fit is not significant, *P*‒value = 0.1464). The summary statistics of the quadratic model showed the smallest standard deviation of 0.69 and the largest adjusted and predicted R-squared of 0.9792 and 0.9340; respectively.

The coefficients of regression equation were calculated and the data (Table 4) was fitted to a second-order polynomial equation. The Pb^2+^ removal (Y) by *Gelidium amansii* biomass can be expressed in terms of the following regression equation:

2 $$\begin{array}{rcl}Y & = & +99.80+0.43\,{{\rm{X}}}_{1}+0.55\,{{\rm{X}}}_{2}-0.65\,{{\rm{X}}}_{3}+0.26\,{{\rm{X}}}_{1}{{\rm{X}}}_{2}-1.40\,{{\rm{X}}}_{1}{{\rm{X}}}_{3}\\  &  & +2.29\,{{\rm{X}}}_{2}{{\rm{X}}}_{3}-2.63\,{{{\rm{X}}}_{1}}^{2}\mbox{--}1.86\,{{{\rm{X}}}_{2}}^{2}-4.36\,{{{\rm{X}}}_{3}}^{2}\end{array}$$

where Y is the predicted value of Pb^2+^ removal %, X_1_, X_2_ and X_3_ are the coded levels of initial pH, Pb^2+^ concentration, and temperature.

### Contour and three dimensional (3D) surface plots

To explain the relationship between each pair-wise combination of the three variables (X_1_X_2_, X_1_X_3_ and X_2_X_3_) and the responses, three-dimensional and corresponding contour plots were generated by plotting the response (Pb^2+^ removal %) on Z-axis against two independent factors while keeping the other factor at its central point (zero level) to determine the optimum conditions for Pb^2+^ removal %.

**Figure 3:**
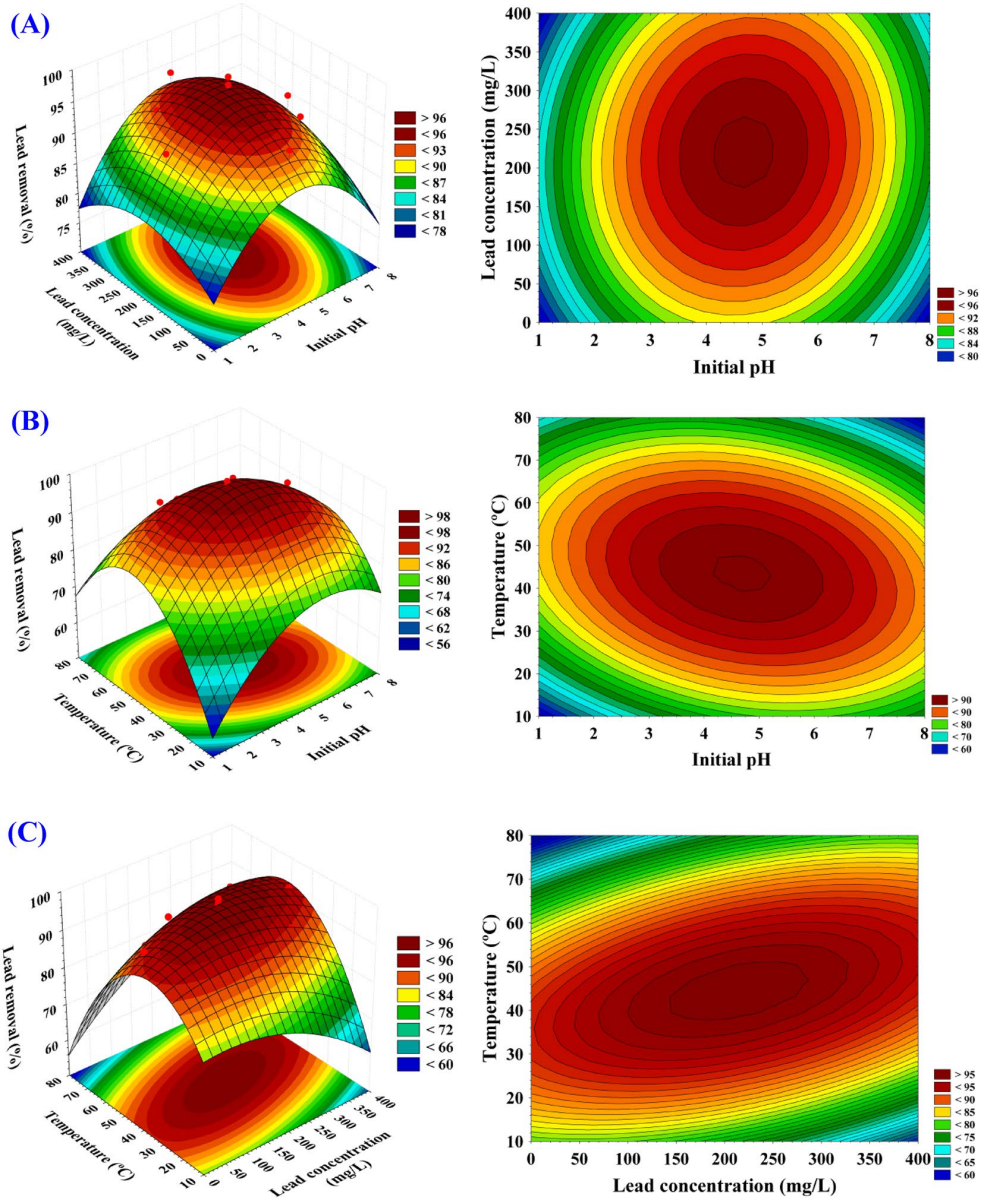
3D response surface and contour plots of the effects of pH (X_1_), Pb^2+^ concentration (X_2_) and temperature (X_3_) and their mutual effects on Pb^2+^ removal by *Gelidium amansii* biomass.

The three dimensional surface plot and its corresponding contour plot (Fig. 3A), showing the simultaneous effect of initial pH (X_1_) and Pb^2+^ concentration (X_2_) on Pb^2+^ removal %, while temperature (X_3_) was kept at their zero levels (45 °C). Figure 3A shows that the lead removal % increased with an increase in the initial pH up to a certain pH value and then further increase in pH resulted in a gradual decrease in Pb^2+^ removal percentage. On the other hand, it can be seen from Fig. 3A that the lead removal % increased with increase in Pb^2+^ concentration (X_2_) and higher levels of Pb^2+^ concentration support relatively low percentage of lead removal. By solving the Eqn. (2) and analysis of Fig. 3A, the maximum predicted Pb^2+^ removal percentage of 99.85% was obtained at the optimum predicted levels of pH and Pb^2+^ concentration of 4.6 and 211 mg/L; respectively at temperature of 45 °C.

Figure 3A shows that the effect of initial lead ions concentration was significant and had a positive effect on biosorption of metal ions. The biosorption of lead ions increased with increasing lead ions concentration and reached the maximum. However, further increasing in lead concentration leads to a gradual decrease in the removal percentage. This can be attributed to that the adsorption sites on the surface area of algal biomass were free and remain unsaturated at the beginning, resulting in high metal adsorption. Thereafter, with increasing metal concentration, the metal removal percentage decreased due to the adsorption sites on the surface of algae were saturated^39^. Increasing initial lead ion concentration increased lead removal by the adsorbent, could be attributed to the increase in the mass transfer due to higher driving force^40,41^.

Figure 3A shows that the biosorption of Pb^2+^ on the surface of *Gelidium amansii* is highly pH dependent. Numerous studies show that pH is an important factor affecting heavy metals biosorption^42^. In the biosorption process, it is well known that pH could affect the functional groups and the metal binding sites on the cell surface of the biomass as well as the metal ion solubility in water^43^. The cell walls of red algae possess complex cell walls composed of cellulose, xylan or mannan fibrils and extensive matrix polysaccharides including the economically important carrageenan, agar, gelans (mucusy sugars) and proteins together with minerals. Some red algae can absorb calcium from seawater and store calcium carbonate in their bodies^44,45^. These components can provide different functional groups as binding sites for the metal ions. At low pH, the protons of functional groups give a generally positive charge to the polymer molecules that are unable to adsorb positively charged heavy metal ions. Increasing the pH reduces the electrostatic repulsion, exposing more ligands on the alga cell wall carrying negative charges such as amino, phosphate and carboxylic groups with subsequent attraction and biosorption of metal ions^46,47^. The functional groups such as hydroxyl and amino groups were found to be responsible for the biosorption of lead ions. This is evidence that the lead biosorption occurs through the ion exchange mechanism where the lead ions is linked to the binding sites by replacing two acid H at low pH^48^. The increase in availability of the adsorption sites improved the access of lead to the adsorption sites of the adsorbent^49^. Moreover, the pH value affects the solubility of the metal ions in solution^50^. Lead is present in its free ionic forms (Pb^2+^) at pH values less than 5. The significant increase of the biosorption of metal ions by increasing the pH to 4.5 is may be due to the cell walls would have a negative net charge, which promotes electrostatic attractions between positively charged Pb^2+^ cations and negatively charged binding sites. Thus, higher pH value may affect the number of negatively charged sites, which is highly dependent on the dissociation of functional groups. In addition, H^+^ competes with Pb^2+^ for the same adsorption position^24,51^. The decrease in the biosorption of metal ions at higher pH values could be related to the repulsion between the negative charge of anionic species in solution and negative surface charge of the sorbents^52,53^. Also, the precipitation of insoluble metal hydroxides occurs restricting the biosorption process. High alkaline pH causes decrease in the solubility of metals, which causes a decrease in absorption rate^54^. Therefore, the best removal occurs at a pH that ranged from 3 to 5. At pH below 2.5, the positive charge (H^+^) density on the sites of biomass surface minimizes metal sorption, and above 6, metal precipitations is favored^55^. The maximum removal of lead on biosorbent by chemically-modified biomass of marine brown alga *Laminaria japonica* was observed at pH 5.3^56^.

The 3D surface plot and its corresponding contour plot in Fig. 3B shows Pb^2+^ removal efficiency as function of initial pH (X_1_) and temperature (X_3_) while Pb^2+^ concentration (X_2_) was kept at their zero levels (200 mg/L). It is evidence from Fig. 3B that the Pb^2+^ removal increased at pH beyond 4.5 after which Pb^2+^ removal decreased. Lower and higher levels of temperature (X_3_) support relatively low percentage of Pb^2+^ removal and the maximum percentage of Pb^2+^ removal clearly situated close to the central point of temperature. By solving the Eqn. (2) and analysis of Fig. 3B, the maximum predicted Pb^2+^ removal of 99.84% was obtained at the optimum predicted levels of pH and temperature of 4.7 and 43 °C; respectively at Pb^2+^ concentration of 200 mg/L.

Figure 3B shows that with an increase in temperature, the biosorption of lead ions by *Gelidium amansii* increases. The effect of temperature on the biosorption process found in the literature presents different and opposite behaviours. Patel and Chandel^57^, Córdova *et al*.^58^ and Rathinam *et al*.^59^ reported higher uptake capacities in different organisms as temperature increases. On the other hand, Ho *et al*.^60^ and Dal Bosco *et al*.^61^ reported practically temperature-independent effect on biosorption capacity. In contrast, Cruz *et al*.^62^ and Aksu^63^ obtained a decrease in the uptake capacity with temperature increase. A similar trend to our results was obtained by previous studies. The maximum Pb(II) removal rate by algae was found to increase with an increase in temperature and reached the maximum value (98%) at the temperature of 40 °C^64^. This means that the binding of Pb (II) on the active sites of the biosorbent becomes stronger at a higher temperature and that the biosorption process is endothermic. The most suitable sorption temperature for the removal of Pb^2+^ and Cd^2+^ in aqueous effluent using *Caladium bicolor* biomass was obtained at 40 °C^65^. The optimal temperature for completely lead removal from aqueous solutions with *Aspergillus terreus* was 50 °C^58^. Patel and Chandel^57^ reported that 94% Cu ions was removed at 45 °C using *Bacillus licheniformis*. Rathinam *et al*.^59^ reported that the maximum cadmium biosorption from simulated wastewaters has been obtained at 60 °C using red alga *Hypnea valentiae* biomass.

The increase in biosorption rate can be attributed to the increase in temperature is known to increase the ions diffusion rate of adsorbed molecules from the aqueous solution to the biosorbent surface as a result of the reduced viscosity of the solution^66,67^. Higher temperatures usually enhance sorption due to the increased surface activity and kinetic energy of the solute^28^. The increase in biosorption rate may be due to the formation of new adsorption active sites^68^. Saleem *et al*.^69^ reported that with an increase in temperature, the pores of the algae biomass surface enlarge resulting in an increase of the surface area available for the sorption, diffusion and penetration of the metal ions within the pores of surface resulting in an increase in biosorption. Only a small increase in cadmium biosorption by marine macroalga *Cystoseira baccata* as biosorbent was obtained at 45 °C^70^. The alginate chains in brown algae yield an array of cavities known as the egg-box structure^71^ which may stabilize the alga biomass at higher temperature and increase metal biosorption.

However, further increase in temperature above 45 °C results in a decrease in the removal efficiency that can be attributed to deactivating the biosorbent surface, or destructing of some active sites on the biosorbent surface due to rupture of the bonds^72^ or due to the weakness of biosorption forces between the active sites on the surface of the algae and the lead ions. Physical damage to the biosorbent can be expected at higher temperatures^28^.

The 3D plot and its corresponding contour plot (Fig. 3C), shows the effects of Pb^2+^ concentration (X_2_) and temperature (X_3_) on Pb^2+^ removal efficiency, when pH (X_1_) was kept at their zero levels (4.5). The effect of temperature is found to be more significant than that of the initial Pb^2+^ concentration. The percentage Pb^2+^ removal increased with increasing both of initial Pb^2+^ concentration and temperature to the optimum levels and thereafter the Pb^2+^ removal decreased. By solving the Eqn. (2) and analysis of Fig. 3C, the maximum predicted Pb^2+^ removal of 99.84% was obtained at the optimum predicted levels of Pb^2+^ concentration and temperature of 206 mg/L and 44 °C; respectively at pH 4.5.

According to the results of RCCD, the maximum removal percentage of Pb^2+^ from aqueous solution by *Gelidium amansii* biomass (100%) was found under the optimum conditions: initial Pb^2+^ concentration of 200 mg/L, temperature 45 °C, pH 4.5, *Gelidium amansii* biomass of 1 g/L and contact time of 60 minutes at static condition. The maximum bioremediation efficiency of 90% of Pb^2+^ in optimal conditions by using red alga *Porphyra leucosticta* was at biomass dosage 15 g/L, pH 8 and contact time 120 minutes containing initial 10 mg/L of Pb^2+^ solution^73^. The optimum biosorption conditions of lead (II) ions on *Sargassum ilicifolium*, brown seaweed, were determined as initial pH 3.7, biosorbent concentration 0.2 g/L, and initial Pb^2+^ ion concentration 200 mg/L^74^.

Under optimum conditions, the maximum biosorption of Pb^2^ using *Cystoseira trinodis* (brown algae) was found to be 49.08 mg/g. These conditions were a pH of 5.2, initial Pb^2+^ ion concentration of 200 mg/L and a contact time of 60 minutes^75^. Rajasimman and Murugaiyan^76^ reported that the maximum removal of lead from aqueous solution on *Hypnea valentiae*, red macro algae, was found to be 91.97% at the optimum conditions for the sorption pH: 5.1, sorbent dosage: 5.1 g/L, temperature: 36.8 °C, contact time: 30 minutes and metal concentration: 100 mg/L. The feasibility of *Spirulina maxima* was studied as biosorbent for Pb^2+^ removal from aqueous solution. The biosorption was pH dependent and the maximum ratio of lead adsorption was about 84% was obtained at pH value of about 5.5 for 60 minutes^77^. The optimum conditions for lead biosorption by non-living (dried) fresh water algae, *Oedogonium* sp. and *Nostoc* sp. are almost same for the two algal biomass (pH 5.0, contact time of 90 and 70 minutes, biosorbent dose of 0.5 g/L and initial Pb^2+^ concentration 200 mg/L)^24^.

### Verification of the model

According to second-order polynomial models, the maximum removal percentage of Pb^2+^ from aqueous solution by *Gelidium amansii* biomass was found under the optimum conditions: initial Pb^2+^ concentration of 200 mg/L, temperature 45 °C, pH 4.5, *Gelidium amansii* biomass of 1 g/L and contact time of 60 minutes at static condition. Under these conditions, the maximum Pb^2+^ removal percentage of 100% was verified and compared with the predicted value from the polynomial model (99.8%). The verification showed a high degree of accuracy of the model, demonstrating the model validation under the concentrations used.

### FTIR analysis

**Figure 4:**
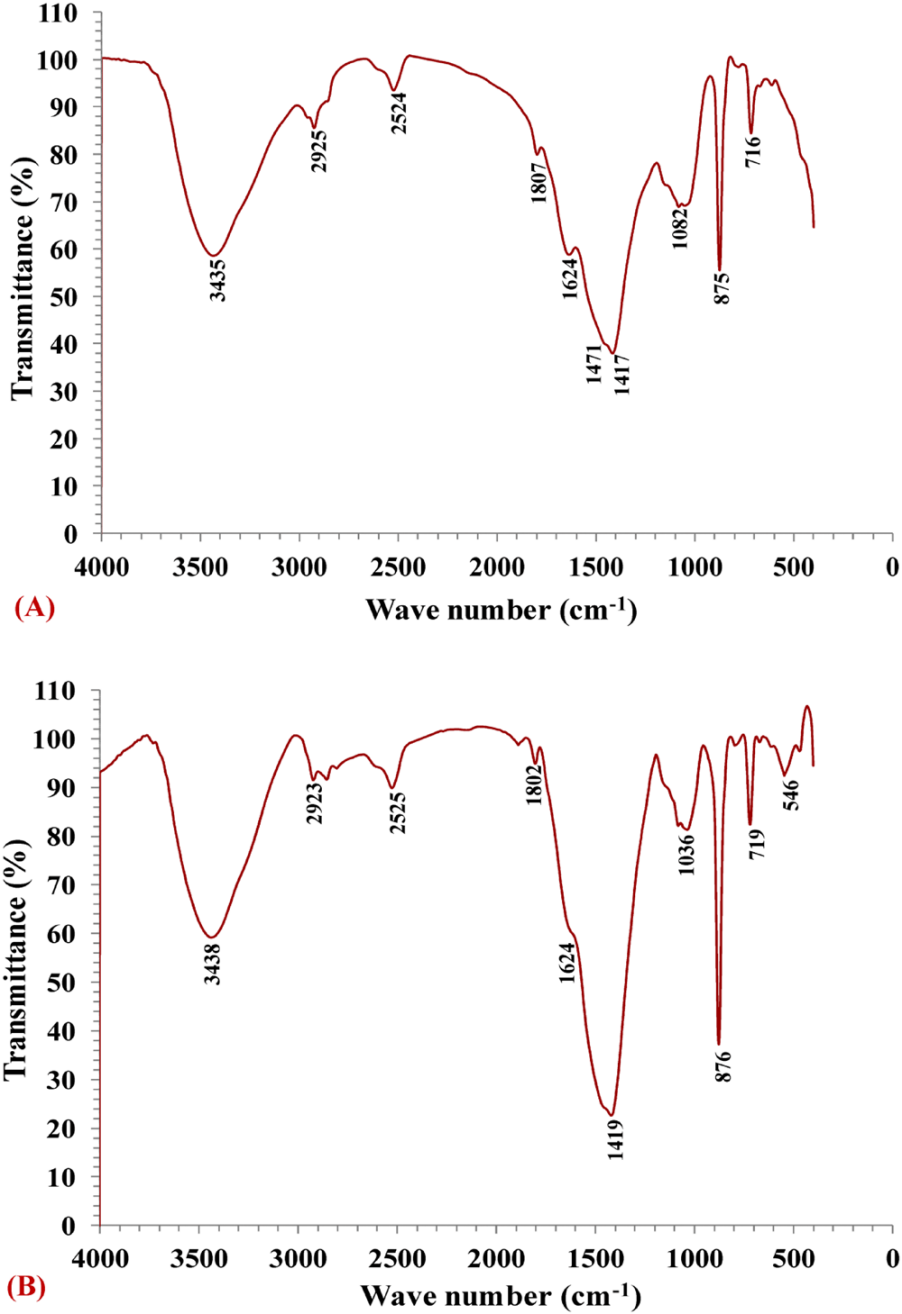
FTIR of *Gelidium amansii* biomass: (**A**) before Pb^2+^ ions biosorption; (**B**) after Pb^2+^ ions biosorption from aqueous solution.

The FTIR spectrums of *Gelidium amansii* biomass samples were analyzed before and after Pb^2+^ biosorption (Table 6 and Fig. 4) to detect any differences due to the interaction between the functional groups on the *Gelidium amansii* biomass and Pb^2+^ ions during biosorption process. The cell walls of red algae generally contain cellulose and sulfated polysaccharides (carrageenan and agar)^78^. The carrageenan corresponds up to 75% of the dry weight of the biomass^79^. The carboxylic groups are the most abundant acidic functional group and the adsorption capacity of algae is directly attributed to the presence of these binding sites. The red algae are mainly composed of carrageenan that provides different binding sites (e.g., hydroxyl, carboxyl, amino and sulfhydryl) responsible for Pb^2+^ ions biosorption.

**Table 6:** Analysis of FTIR spectrum results of *Gelidium amansii* biomass before and after Pb^2+^ ions biosorption from aqueous solution.

Before Pb^2+^ ions biosorption		After Pb^2+^ ions biosorption		Difference
Wave number (cm^−1^)	Annotations	Wave number (cm^−1^)	Annotations	
3435	O–H group	3438	O–H group	3
2925	CH_2_ group	2923	C–H stretching vibration	−2
2524	S–H stretching vibration	2525	S–H stretching vibration	1
1807	Carbonyl group (C=O) stretching vibration	1802	C=C bond stretching vibration	−5
1624	C=O stretching vibration of the ketone	1624	C=O stretching vibration of the ketone	0
1471	Vibration of the CH_2_ group	1419	Carboxyl COO− units	−52
1417	Carbonyl group, C=O	1036	C‒O and C‒O‒C stretching vibrations	−381
1082	PO_2_–vibrations of phospholipids	876	C=O stretching vibration	−206
875	CO_3_ vibrations of calcite	719	C–H bend of alkene	−156
716	C‒O‒C bending vibration	546	Vibration of P=O in PO_4_^3−^	−170

FTIR spectrum for biomass sample before Pb^2+^ biosorption showed the characteristic absorption peaks at 3435, 2925, 2524,1807, 1624, 1471, 1417, 1082, 875 and 716 cm^−1^ were shifted to 3438, 2923, 2525, 1802, 1624,1419, 1036, 876, 719 and 546 cm^−1^; respectively after lead biosorption by the biomass. These changes in the wave numbers and their intensity were as a result of the interaction between the functional groups on the *Gelidium amansii* biomass and Pb^2+^ ions during biosorption process. The broad peak observed at 3435 cm^−1^ in biomass sample before lead biosorption is assigned to the stretching of O–H group due to molecular hydrogen bonding of polymeric compounds, such as alcohols, phenols and carboxylic acids. The peak at 3438 cm^−1^ is assigned to O–H groups^80^. The O–H stretching vibrations occur within a broad range of frequencies indicating the presence of free hydroxyl groups and bonded O–H peaks of carboxylic acids^81,82^. The peak at 2925 cm^−1^ is attributed to the symmetric stretching vibration of the aliphatic CH_2_ group^83^. While, the peak at 2923 cm^−1^ is attributed to C–H stretching vibration belonging to lipids and phospholipids fractions^84^. The minor peak at 2524 cm^−1^, corresponding to the S–H stretching vibration mode^85^. Moreover, the peak at 2525 cm^−1^, assigned to the S–H vibration^86^.

The FTIR spectrum showed a peak at 1807 cm^−1^ corresponding to the stretching vibrations of the carbonyl group (C=O)^87^. The absorption peak at 1802 cm^−1^ corresponds to C=C bond stretching vibration^88^. The peak at 1624 cm^–1^ is assigned to the C=O stretching vibration of the ketone^89^ or due to the stretching vibrations of C=C^90^. The peak at 1471 cm^−1^ in algal biomass before biosorption of Pb^2+^ was consistent with the bending vibration of the CH_2_ group^91^ is shifted by −52 cm^−1^ to the intense peak at 1419 cm^−1^ which result due to absorption from carboxyl COO–units^92^. Furthermore, the interaction between the algal biomass and Pb^2+^ during biosorption process included a large up shift of the peak at 1417 cm^−1^ to 1036 cm^−1^ by −381 cm^−1^. The peak at 1417 cm^–1^ is strongly associated with the presence of carbonate minerals (correlated to carbonyl group, C=O)^93^. The peak at 1036 cm^−1^ represents the C=O stretching region as complex peaks resulting from C=O and C‒O‒C stretching vibrations, indicating the presence of carbohydrate content in the sample^94^. Also, the interaction between the algal biomass and Pb^2+^ during biosorption process included a large up shift of the peak at 1082 cm^−1^ to sharp peak at 876 cm^–1^ by −206 cm^−1^. The peak at 1082 cm^−1^ was attributed to PO_2_ − asymmetric and symmetric stretching vibrations of phospholipids^95^. The presence of sharp peak at 876 cm^−1^ is due to the C=O stretching vibration^96^. The absorption peak at 875 cm^−1^ wave number in algal biomass before biosorption of Pb^2+^ is the characteristic absorption peak of CO_3_ vibrations of calcite^97^ is shifted by −156 cm^−1^ to the peak observed at 719 cm^−1^ which corresponds to C–H bend of alkene^98^. Furthermore, IR spectral data revealed a shift in peak position from716 cm^−1^ in algal biomass before biosorption of Pb^2+^ to 546 cm^−1^ after Pb^2+^ biosorption. The peak at 716 cm^−1^ is associated with the C‒O‒C bending vibration separately in glycosidic linkages^99^ while the peak at 546 cm^−1^ assigned to asymmetric deformation vibration of P=O in PO_4_^3−^^100^.

In conclusion, FITR confirmed that the carboxyl, carbonyl, methylene, phosphate, carbonate, and phenolic groups were the main groups involved in the Pb^2+^ ions biosorption process. The carboxyl and amino functional groups provide the major biosorption sites for the lead binding. Other functional groups such as alcoholic groups also have an important role in metal uptake^101^.

### Scanning electron microscopy (SEM)

**Figure 5:**
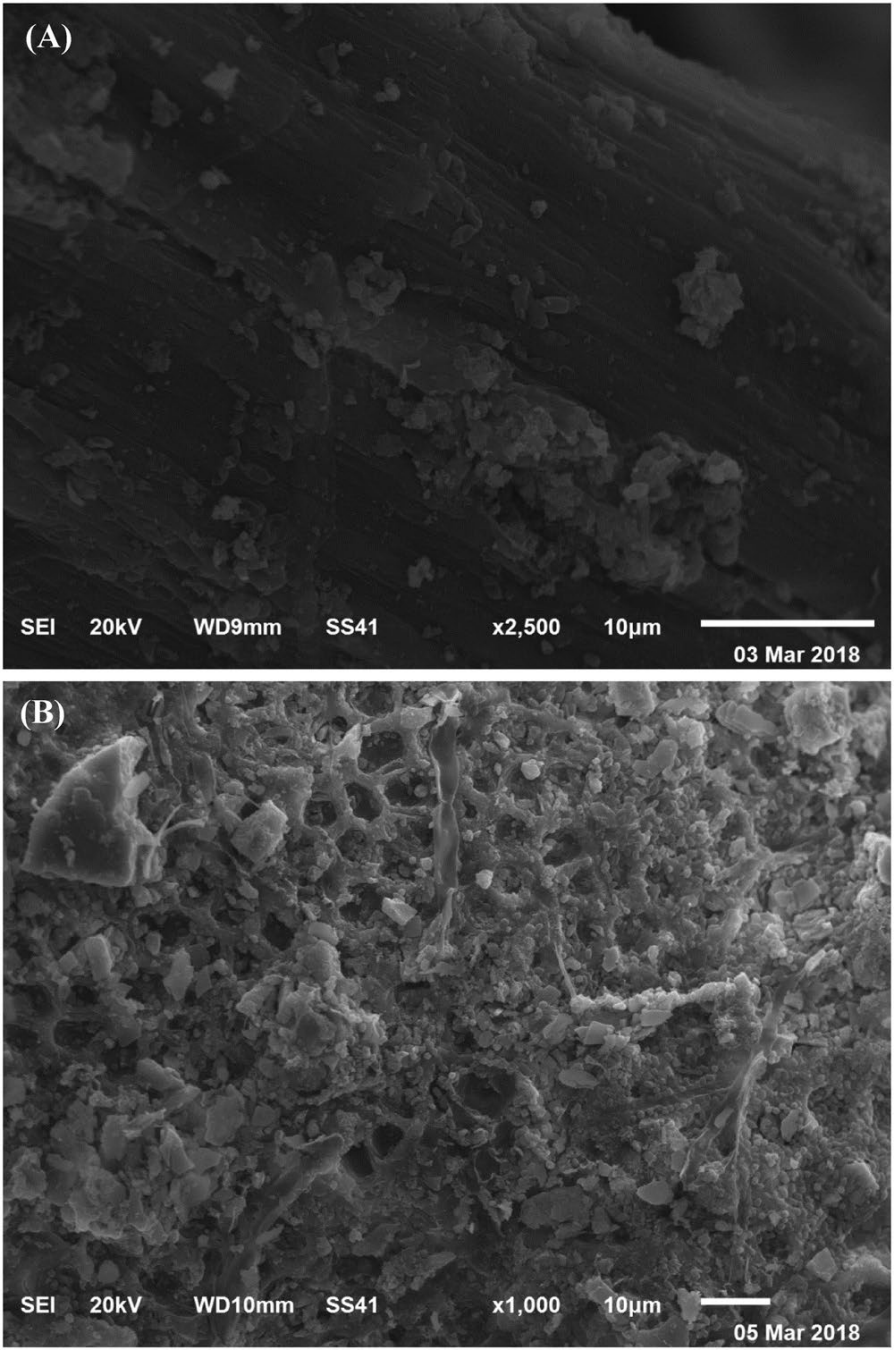
SEM micrograph of *Gelidium amansii* biomass: (**A**) before and (**B**) after adsorption of Pb^2+^ ions from aqueous solution.

SEM is used to verify the morphological differences between the *Gelidium amansii* biomass before and after adsorption of Pb^2+^ ions. Figure 5A shows SEM micrograph of *Gelidium amansii* biomass before adsorption of Pb^2+^ ions and Fig. 5B shows SEM micrograph of *Gelidium amansii* biomass after adsorption of Pb^2+^ ions. SEM images have clearly shown that the dry *Gelidium amansii* biomass samples before and after Pb^2+^ biosorption exhibited different surface morphologies. As shown in Fig. 5A *Gelidium amansii* biomass exhibited uniform interconnected structure with a continuous surface. Graph in Fig. 5B demonstrate the ability of *Gelidium amansii* biomass to adsorp and remove Pb^2+^ from aqueous solutions. After Pb^2+^ biosorption, the walls of biomass have become fragile, irregular surface with the appearance of bright spots due to the accumulation of Pb^2+^.

### Electron dispersive spectroscopy (EDS)

**Figure 6:**
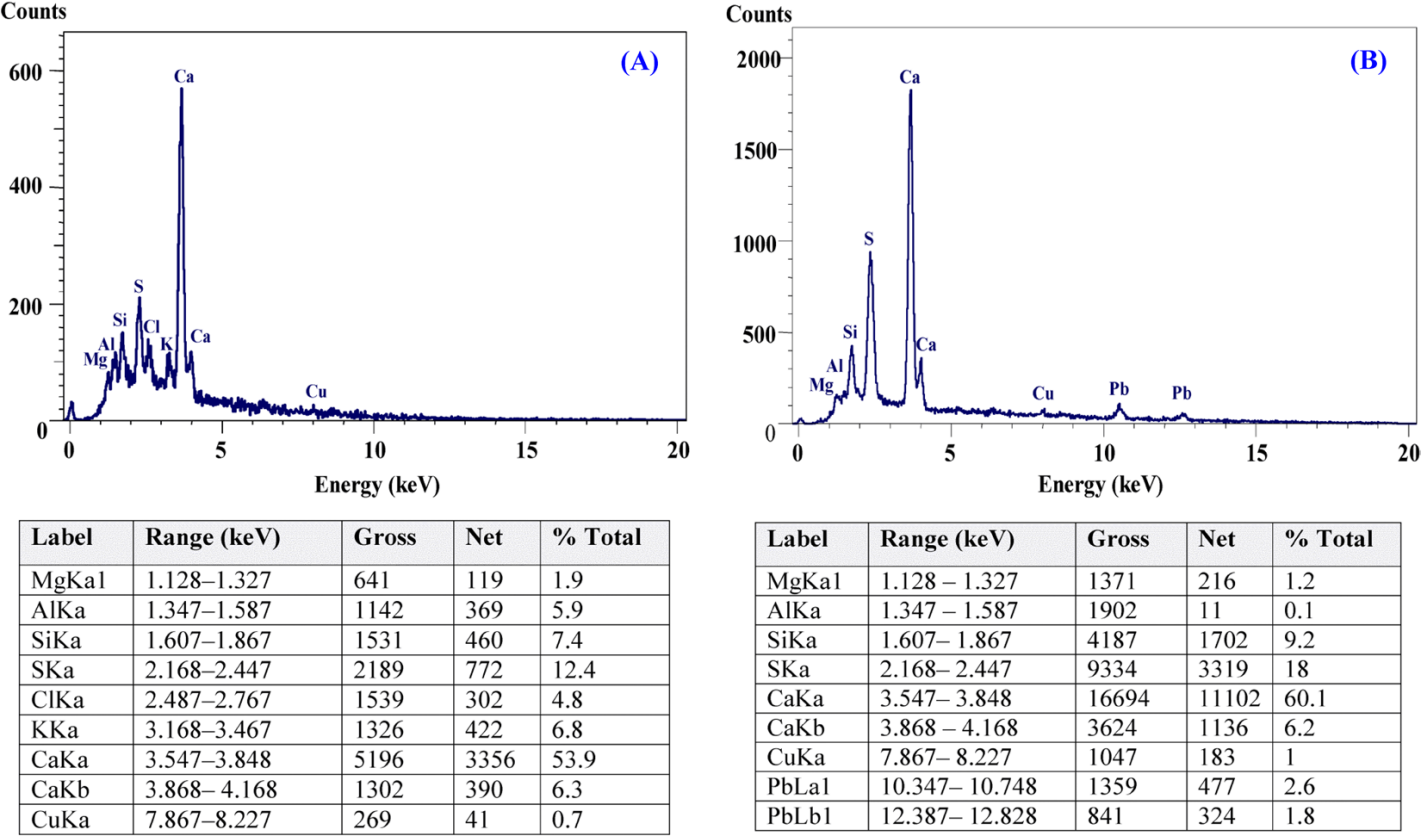
EDS analysis for *Gelidium amansii* biomass (**A**) before Pb^2+^ ions biosorption; (**B**) after Pb^2+^ ions biosorption from aqueous solution.

EDS is a useful tool used for chemical characterization or the elemental analysis of biosorbents^102^. In the present study, EDS analysis was performed to find out type of the elements present in the sample and confirmation of the presence of Pb^2+^ attached to the cell surface of *Gelidium amansii* biomass. Figure 6B shows the additional optical absorption peak corresponding to the Pb^2+^ is detected in the biomass after adsorption of Pb^2+^ ions which confirms the involvement of *Gelidium amansii* biomass in the adsorption of Pb^2+^ ions from aqueous solution. Kim *et al*.^103^ reported that after the contact with lead, the characteristic lead peak was appeared.

### Immobilization of *Gelidium amansii* in alginate beads and its application in Pb^2+^ removal

**Figure 7:**
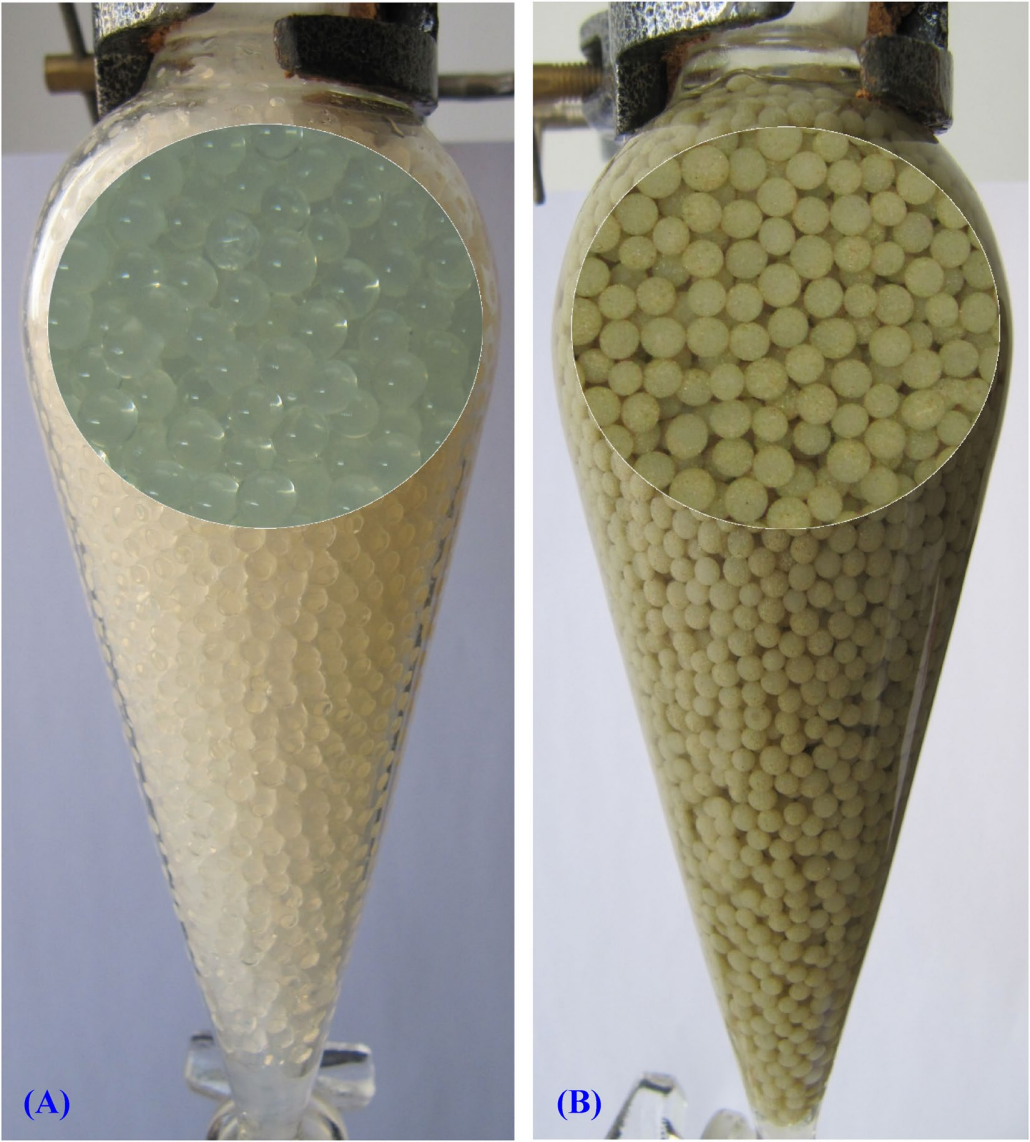
Immobilization of *Gelidium amansii* biomass in alginate beads and its application in Pb^2+^ removal from aqueous solution. (**A**) Sodium alginate beads without incorporation of the algal biomass; (**B**) Separating funnel packed with immobilized *Gelidium amansii* biomass in sodium alginate beads.

**Figure 8:**
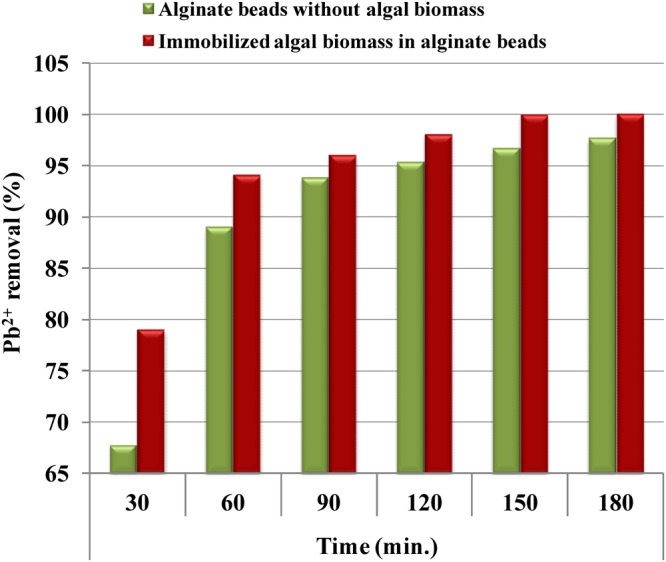
Application of immobilized *Gelidium amansii* biomass in Pb^2+^ removal from aqueous solution.

The ability to remove Pb^2+^ in aqueous solution by immobilized *Gelidium amansii* biomass in sodium alginate beads was studied (Fig. 7) and the results are presented in Fig. 8. The results indicated that the treatment of aqueous solution containing Pb^2+^ with immobilized *Gelidium amansii* biomass in sodium alginate-beads removed 100% of Pb^2+^ at an initial concentration of 200 mg/L for 3 h, which is significantly higher than the removal presented using sodium alginate beads without incorporation of the algal biomass as a control (97.68%) (Fig. 8). Some studies reported that immobilized biomass has the potential to provide a simple technology to remove and recover heavy metals from wastewater, and is suitable for reuse compared to free cells^104,105^. The size of the bead used for immobilization of biomass is an important factor^106^. The removal efficiency of Pb^2^ by immobilized *Microcystis aeruginosa* reached 80% for Pb^2+^^107^. Abdel Hameed^108^ reported that the efficiency of the immobilized beads over the free cells. The high lead removal by the immobilized beads of *Chlorella vulgaris* was 92%. However, lead removal was mainly caused by the alginate beads matrix with only a slight contribution by *Chlorella vulgaris*. Immobilization tends to increase the accumulation of metal by biomass^4^. Immobilized cells more effective than free cells for metal removal by biomass due to increase in the cell wall permeability^109^.

## Material and Methods

### Collection of marine alga and biomass preparation

*Gelidium amansii* is a red alga, was collected from Abukir beach, Alexandria governorate, Egypt. For removal of the external sand and salts, the collected biomass of *Gelidium amansii* was washed with tap water and then twice with distilled water. The algal biomass was dried in oven at 70 °C for 72 hours, and then grinded with a blender, sieved to get particle with the size pass through a laboratory test sieve (Endecotts/ Ltd., London, England) with mesh size of 125 µm. 20 g of the dried biomass of *Gelidium amansii* were thoroughly mixed with 1 L of distilled in 2 L Erlenmeyer flask and the suspension mixed, incubated at room temperature under stirring for about 30 minutes. Then, the homogeneous suspension of *Gelidium amansii* biomass was filtered and dried at 70 °C for 72 hours or until constant weight was obtained and kept at room temperature for further use in biosorption experiments.

### Preparation of lead solution

Pb^2+^ solutions were prepared by dissolving lead acetate (Pb(CH_3_COO)_2_.3H_2_O) in 100 mL of distilled water to attain the needed concentrations (25‒368.18 mg/L). The initial pH of each solution was adjusted to the required value with 0.1 N H_2_SO_4_ and 0.1 N NaOH.

### Selection of significant variables for Pb^2+^ removal by Plackett–Burman design

The Plackett–Burman Design (PBD)^110^ is an efficient screening method to detect the significant variables among large number of variables that influences a process^111,112^. PBD was used for the selection of the variables that had a significant effect, either positively or negatively on Pb^2+^ biosorption out of six variables. The six variables (independent variables) including: different contact times (60 and 180 minutes), Pb^2+^ ions concentration (25 and 200 mg/L), two different initial pH levels (4 and 7) which was adjusted with 0.1 N H_2_SO_4_ and 0.1 N NaOH, temperature (25 and 50 °C), biomass concentration (1 and 4 g/L) and static or agitation condition. Each variable was examined in two levels, low (−) and high (+) level. 12 runs Plackett–Burman design was used to evaluate the effect of the selected six variables on the Pb^2+^ removal efficiency. In the experimental design each row represents an experiment and each column represents an independent variable (Table 1).

Plackett–Burman experimental design is based on the first order model equation:

3 $${\boldsymbol{Y}}={{\boldsymbol{\beta }}}_{{\boldsymbol{0}}}+\sum {{\boldsymbol{\beta }}}_{{\boldsymbol{i}}}{{\boldsymbol{X}}}_{{\boldsymbol{i}}}$$

where, Y is the measured response (Pb^2+^ removal %), β_0_ is the model intercept and *β*_*i*_ is the linear coefficient, and X_i_ is the level of the independent variable.

Dry biomass of *Gelidium amansii* was thoroughly mixed with the solution of Pb^2+^ in Erlenmeyer flasks. The suspensions were kept static or with agitation for specific contact time at the selected temperature.

### Pb^2+^ quantification by ICP-AES

The solutions were filtered through disposable 0.2 µm PTFE syringe filters (DISMIC-25HP, Advantec, Tokyo, Japan). The residual Pb^2+^ concentrations in the solutions were determined by means of inductively coupled plasma–atomic emission spectroscopy (ICP-AES, Thermo Scientific, Germany). Certified reference materials (Merck, Germany) were included in the analyses. The recovery of metals was within the certified limits as 10 ppb to 1000 ppb. To get the final concentration, the solution was diluted with 0.1 mM H_2_NO_3_ and the final dilution factors were used^113^.

All experiments were carried out in triplicate and determination of Pb^2+^ removal is average of three trials.

### Optimization of Pb^2+^ removal by rotatable central composite design (RCCD)

Based on the results of PBD, a three factor, five levels rotatable central composite design was performed to determine the optimum levels of the significant variables and the individual and interactions between the selected variables with high influence on Pb^2+^ removal. The three factors selected from PBD, for further optimization using RCCD were pH, Pb^2+^ concentration (mg/L), and temperature (°C) which were denoted as X_1_, X_2_ and X_3_; respectively. Rotatable CCD had twenty different experiments with six center points was generated with Design Expert version 7 for Windows software. The significant variables were assessed at five coded levels (−1.68, −1, 0,+1 and +1.68), as is shown in Table 3. Linear, quadratic and interaction effects of the three variables on Pb^2+^ removal were calculated. The relationship between the Pb^2+^ removal (Y) viz the significant independent variables (X_1_, X_2_ and X_3_) is given using the following second order polynomial equation:

4 $${\boldsymbol{Y}}={{\boldsymbol{\beta }}}_{{\boldsymbol{0}}}+\sum _{{\boldsymbol{i}}}{{\boldsymbol{\beta }}}_{{\boldsymbol{i}}}{{\boldsymbol{X}}}_{{\boldsymbol{i}}}+\sum _{{\boldsymbol{ii}}}{{\boldsymbol{\beta }}}_{{\boldsymbol{ii}}}{{\boldsymbol{X}}}_{{\boldsymbol{i}}}^{2}+\sum _{{\boldsymbol{ij}}}{{\boldsymbol{\beta }}}_{{\boldsymbol{ij}}}{{\boldsymbol{X}}}_{{\boldsymbol{i}}}{{\boldsymbol{X}}}_{{\boldsymbol{j}}}$$

In which Y is the predicted Pb^2+^ removal, β_0_ is the regression coefficients, β_i_ is the linear coefficient, β_ii_ is the quadratic coefficients, β_ij_ is the interaction coefficients, and X_i_ is the coded levels of independent variables.

Three additional confirmation trials were performed to verify the accuracy of the statistical experimental design.

### Statistical analysis of the data

Minitab and Design Expert version 7 for Windows softwares were “used for the experimental designs and statistical analysis. The regression analysis of the obtained experimental data was performed to calculate the analysis of variance (ANOVA). The percentage of contribution of each variable was calculated. The statistical software package, STATISTICA software (Version 8.0, StatSoft Inc., Tulsa, USA) was used to plot the three-dimensional surface plots”. The response surface and contour plots were used to assess the relationship between the significant variables.

### Analytical methods

10 mL of filtrate from each trial filtered through disposable 0.2 µm PTFE syringe filters (DISMIC-25HP, Advantec, Tokyo, Japan) and analyzed using inductively coupled plasma – atomic emission spectroscopy (ICP-AES, Thermo Scientific. The efficiency of *Gelidium amansii* biomass for Pb^2+^ ions removal from aqueous solutions was calculated quantitatively by using the following equation:

5 $${\bf{Removal}}\,{\bf{efficeincy}}\,{\boldsymbol{(}}{\boldsymbol{ \% }}{\boldsymbol{)}}=\frac{{{\bf{C}}}_{{\bf{i}}}-{{\bf{C}}}_{{\bf{f}}}}{{{\bf{C}}}_{{\bf{i}}}}\times {\bf{100}}$$

where: C_i_ is the initial metal ion concentration (mg/L), C_f_ is the final (residual) metal ion concentration (mg/L). All determinations of Pb^2+^ ions in the solution were carried out in triplicates.

### Fourier transform infrared (FTIR) spectroscopy

FTIR analysis was used to confirm the presence of functional groups in the dry *Gelidium amansii* biomass samples before and after Pb^2+^ biosorption. The *Gelidium amansii* biomass samples were incorporated with KBr pellets and the FTIR spectra were measured using Thermo Fisher Nicolete IS10, USA spectrophotometer within the range of 400–4000 cm^−1^.

### Scanning electron microscopy (SEM)

SEM was used to verify the morphological differences between the dry *Gelidium amansii* biomass samples before and after Pb^2+^ biosorption to examine the algal cells surfaces and to evaluate the Pb^2+^ adsorption. The samples were coated with gold and were examined at different magnifications at 20 kV.

### Electron dispersive X- ray spectroscopy (EDS)

EDS helps to find out the type of elements present in the samples. EDS analysis was carried out with “the scanning electron microscope (Oxford X-Max 20) with secondary electron detectors at an operating voltage of 20 kV at Electron Microscope Unit, Faculty of Science, Alexandria University, Alexandria, Egypt”.

### Immobilization of *Gelidium amansii* in alginate beads and its application in Pb^2+^ removal

The biosorption capacity of *Gelidium amansii* biomass for lead ions biosorption from aqueous solution was determined using separating funnel packed with immobilized *Gelidium amansii* biomass in sodium alginate beads. Solution of 4% sodium alginate was prepared by dissolving 4 g sodium alginate (SIGMA-Aldrich) into 100 mL distilled water and mixed thoroughly with continuous stirring for 30 minutes at 60 °C for better dissolution^114^. After cooling, dried and washed 4 g (4%, W/V) of *Gelidium amansii* biomass sieved by laboratory test sieve (125 µm Endecotts/ Ltd., London, England) was added with stirring at room temperature for 5 minutes. The beads of 1.5 mm ± 0.2 mm diameter were obtained by dropping the alginate algal mixture using syringe (3 mL) into a cold sterile 2.5% CaCl_2_ solution in distilled water at room temperature in sterile condition under gentle stirring. The resultant spherical beads were washed several times with autoclaved distilled water to remove unreacted CaCl_2_ from the beads surfaces and then stored overnight at 4 °C in autoclaved distilled water in order to stabilize and harden the beads. By the same procedure, sodium alginate beads without incorporation of the *Gelidium amansii* biomass are also prepared and used as the control. For storage, the beads were dipped in 0.2 M of Tris-HCl buffer (pH 7.2) and stored at 4 °C until further use.

The experiment was conducted in 100 mL separating funnel (Simax glass) packed with alginate algal beads. The solution containing Pb^2+^ ions (200 mg/L) was added to the separating funnel. Samples (5 mL) from the separating funnel effluent were collected regularly (every 30 minutes for up to 3 hours) at a flow rate of 3 mL/minutes and analyzed by inductively coupled plasma – atomic emission spectroscopy (ICP-AES, Thermo Scientific). The biosorption capacity of the Pb^2+^ ions was determined by the difference in Pb^2+^ solution concentration before and after adsorption.

## Conclusion

The potential of *Gelidium amansii* dry biomass for removal of lead ions from aqueous solutions has been investigated in the present study. A two-level Plackett–Burman factorial design was used to determine the most significant variables affecting Pb^2+^ removal %. The most significant variables affecting Pb^2+^ removal chosen for further optimization using rotatable central composite design. The maximum biosorption of Pb^2+^ was 100% at optimum operating conditions: initial Pb^2+^ concentration of 200 mg/L, temperature 45 °C, pH 4.5, *Gelidium amansii* biomass of 1 g/L and contact time of 60 minutes at static condition. Immobilized *Gelidium amansii* biomass was effective in Pb^2+^ removal **(**100%**)** from aqueous solution at an initial concentration of 200 mg/L for 3 h. Based on our results, dry biomass of the red marine alga, *Gelidium amansii*, could be used as a promising, efficient, cheap and biodegradable biosorbent for Pb^2+^ ions removal from wastewater effluents and the process used is safe, feasible and eco-friendly. Also, Plackett–Burman and rotatable central composite designs have been proved to be useful techniques for optimization of the biosorption conditions to get optimum conditions for maximum Pb^2+^ ions removal from aqueous solutions using *Gelidium amansii* dry biomass as adsorbent by significantly reducing the number of experiments, predict the best performance conditions and maintains a good accuracy of the expected response.

## Electronic supplementary material

Supplementary materials
