# Supplementary material for: Biosorption optimization, characterization, immobilization and application of Gelidium amansii biomass for complete Pb2+ removal from aqueous solutions
Source: Sci Rep. 2018 Sep 7;8:13456. doi: 10.1038/s41598-018-31660-7 (PMC6128825; doi:10.1038/s41598-018-31660-7)
Supplement: Supplementary file 1 — Supplementary materials [file 41598_2018_31660_MOESM1_ESM.docx]

**Biosorption optimization, characterization, immobilization and application of *Gelidium amansii* biomass for complete Pb^2+^** **removal from aqueous solutions**

**^1^Noura El-Ahmady El-Naggar, ^2^Ragaa A. Hamouda,** **^3^Ibrahim E. Mousa, ^2^Marwa Salah Abdel-Hamid and ^2^Nashwa H. Rabei**

^1^Department of Bioprocess Development, Genetic Engineering and Biotechnology Research Institute, City of Scientific Research and Technological Applications, Alexandria, Egypt.

^2^Microbial Biotechnology Department, Genetic Engineering and Biotechnology

Research Institute, University of Sadat City, Egypt.

^3^Environmental Biotechnology Department, Genetic Engineering and Biotechnology Research Institute (GEBRI), University of Sadat City, 22857, Egypt.

*To whom correspondence should be addressed.

**Dr. Noura El-Ahmady Ali El-Naggar**

**Address:**

Bioprocess Development Department,

Genetic Engineering and Biotechnology Research Institute,

City of Scientific Research and Technological Applications,

New Borg El- Arab City, 21934, Alexandria, Egypt

**Tel:** (002)01003738444

**Fax:** (002)03 4593423

**E-mail:** [nouraelahmady@yahoo.com](mailto:nouraelahmady@yahoo.com)

**Supplementary Table S1. Fit summary for experimental data**

| **Sequential Model Sum of Squares** | | | | | | |
| --- | --- | --- | --- | --- | --- | --- |
| **Source** | **Sum of Squares** | ***df*** | **Mean Square** | | ***F-*value** | ***P-*value**  ***P*rob >*F*** |
| Linear vs Mean | 12.42 | 3 | 4.14 | | 0.15 | 0.9254 |
| Two factors interaction (2FI) vs Linear | 58.29 | 3 | 19.43 | | 0.68 | 0.5792 |
| Quadratic vs 2FI | 366.08 | 3 | 122.03 | | 253.01 | < 0.0001* |
| Residual | 1.66 | 6 | 0.28 | |  |  |
| **Lack of Fit Tests** | | | | | | |
| **Source** | **Sum of Squares** | ***df*** | **Mean Square** | | ***F-*value** | ***P-*value**  ***P*rob >*F*** |
| Linear | 427.90 | 11 | 38.90 | | 150.81 | < 0.0001* |
| Two factors interaction (2FI) | 369.61 | 8 | 46.20 | | 179.11 | < 0.0001* |
| Quadratic | 3.53 | 5 | 0.71 | | 2.74 | 0.1464 |
| Pure Error | 1.29 | 5 | 0.26 | |  |  |
| **Model Summary Statistics** | | | | | | |
| **Source** | **Standard deviation** | **R-Squared** | | **Adjusted R-Squared** | **Predicted R-Squared** | **PRESS** |
| Linear | 5.18 | 0.0281 | | -0.1541 | -0.4296 | 631.32 |
| Two factors interaction (2FI) | 5.34 | 0.1601 | | -0.2275 | -1.1497 | 949.31 |
| Quadratic | 0.69 | 0.9891 | | 0.9792 | 0.9340 | 29.16 |
| * Significant values,  *df* : degree of freedom, PRESS: sum of squares of prediction error | | | | | | |

**
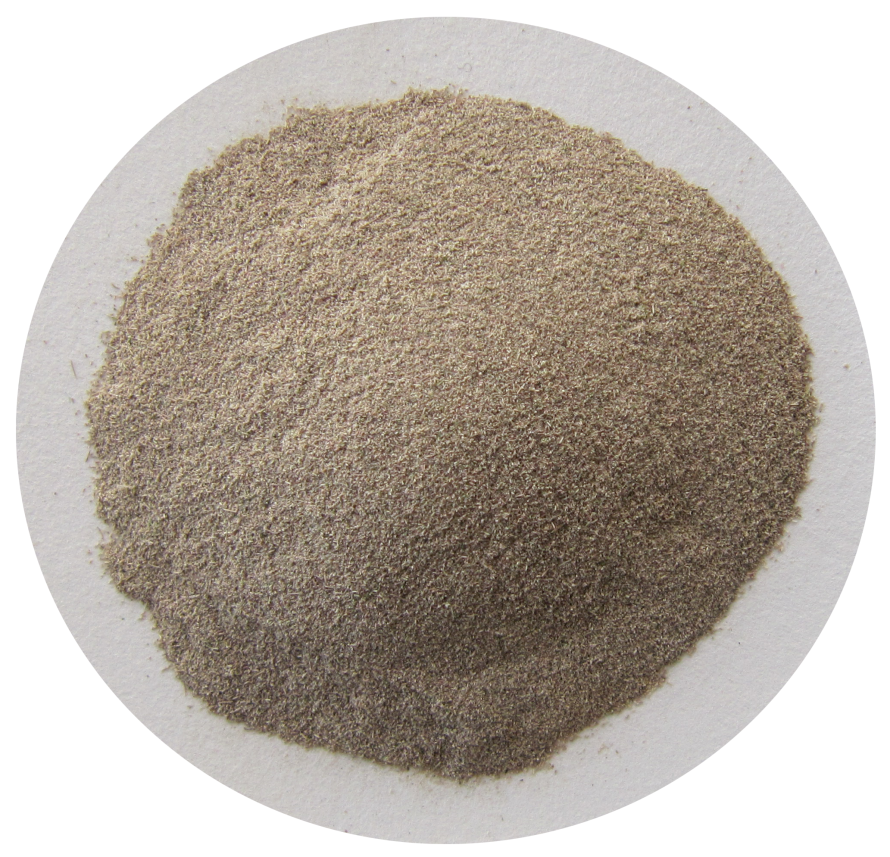
**

**Supplementary Figure S1.** The dried, milled biomass of *Gelidium amansii* with size of 125 µm.

**Supplementary Figure S2.** Pareto chart illustrates the order and significance of the variables affecting Pb^2+^ removal by *Gelidium amansii* biomass using Plackett-Burman design; Ranks (%) values ​​ranging from 1.65 to 48.58).
